# Clearance of Clostridioides difficile Colonization Is Associated with Antibiotic-Specific Bacterial Changes

**DOI:** 10.1128/mSphere.01238-20

**Published:** 2021-05-05

**Authors:** Nicholas A. Lesniak, Alyxandria M. Schubert, Hamide Sinani, Patrick D. Schloss

**Affiliations:** a Department of Microbiology and Immunology, University of Michigan, Ann Arbor, Michigan, USA; Baylor College of Medicine

**Keywords:** 16S rRNA gene, *Clostridioides difficile*, *Clostridium difficile*, colonization resistance, dynamics, microbial ecology, microbiome, microbiota

## Abstract

The gut bacterial community prevents many pathogens from colonizing the intestine. Previous studies have associated specific bacteria with clearing Clostridioides difficile colonization across different community perturbations. However, those bacteria alone have been unable to clear C. difficile colonization. To elucidate the changes necessary to clear colonization, we compared differences in bacterial abundance between communities able and unable to clear C. difficile colonization. We treated mice with titrated doses of antibiotics prior to C. difficile challenge, resulting in no colonization, colonization and clearance, or persistent colonization. Previously, we observed that clindamycin-treated mice were susceptible to colonization but spontaneously cleared C. difficile. Therefore, we investigated whether other antibiotics would show the same result. We found that reduced doses of cefoperazone and streptomycin permitted colonization and clearance of C. difficile. Mice that cleared colonization had antibiotic-specific community changes and predicted interactions with C. difficile. Clindamycin treatment led to a bloom in populations related to *Enterobacteriaceae*. Clearance of C. difficile was concurrent with the reduction of those blooming populations and the restoration of community members related to the *Porphyromonadaceae* and *Bacteroides*. Cefoperazone created a susceptible community characterized by drastic reductions in the community diversity and interactions and a sustained increase in the abundance of many facultative anaerobes. Lastly, clearance in streptomycin-treated mice was associated with the recovery of multiple members of the *Porphyromonadaceae*, with little overlap in the specific *Porphyromonadaceae* observed in the clindamycin treatment. Further elucidation of how C. difficile colonization is cleared from different gut bacterial communities will improve C. difficile infection treatments.

**IMPORTANCE** The community of microorganisms, or microbiota, in our intestines prevents pathogens like C. difficile from colonizing and causing infection. However, antibiotics can disturb the gut microbiota, which allows C. difficile to colonize. C. difficile infections (CDI) are primarily treated with antibiotics, which frequently leads to recurrent infections because the microbiota has not yet returned to a resistant state. The recurrent infection cycle often ends when the fecal microbiota from a presumed resistant person is transplanted into the susceptible person. Although this treatment is highly effective, we do not understand the mechanism. We hope to improve the treatment of CDI through elucidating how the bacterial community eliminates CDI. We found that C. difficile colonized susceptible mice but was spontaneously eliminated in an antibiotic treatment-specific manner. These data indicate that each community had different requirements for clearing colonization. Understanding how different communities clear colonization will reveal targets to improve CDI treatments.

## Abstract (precis)

The community of microorganisms, or microbiota, in our intestines prevents pathogens like C. difficile from colonizing and causing infection. However, antibiotics can disturb the gut microbiota, which allows C. difficile to colonize. C. difficile infections (CDI) are primarily treated with antibiotics, which frequently leads to recurrent infections because the microbiota has not yet returned to a resistant state.

## INTRODUCTION

A complex consortium of bacteria and microbes that inhabits our gut, known as the microbiota, prevents pathogens from colonizing and causing disease. This protection, known as colonization resistance, is mediated through many mechanisms, such as activating host immune responses, competing for nutrients, producing antimicrobials, and contributing to the maintenance of the mucosal barrier (1). However, perturbations to the intestinal community or these functions opens the possibility that a pathogen can colonize (2). For example, the use of antibiotics perturbs the gut microbiota and can lead to Clostridioides difficile infection (CDI).

CDI is especially problematic due to its burden on the health care system (3, 4). C. difficile can cause severe disease, such as toxic megacolon, diarrhea, and death (5). CDI is primarily treated with antibiotics (6). CDIs recalcitrant to antibiotics are eliminated by restoring the community with a fecal microbiota transplant (FMT), returning the perturbed community to a healthier protective state (7, 8). However, FMTs are not always effective against CDI and have the risk of transferring a secondary infection (9, 10). Therefore, we need to better understand how the microbiota clears the infection to develop more effective treatments.

Previous research has shown that the microbiota affects C. difficile colonization. Mouse models have identified potential mechanisms of colonization resistance, such as bile salt metabolism and nutrient competition (11–14). However, studies that have restored those functions were unable to restore complete resistance (15, 16). This could be attributed to the complexity of the community and the mechanisms of colonization resistance (17, 18). We previously showed that when C. difficile colonizes murine communities treated with different antibiotics, it modifies its metabolism to fit each specific environment (14, 19, 20). Therefore, we have investigated the bacterial community dynamics concurrent with clearance of C. difficile below the limit of detection across uniquely perturbed communities.

Jenior et al. (20) observed that clindamycin-treated mice spontaneously cleared C. difficile colonization, whereas mice treated with cefoperazone and streptomycin did not. Here, we continued to explore the different effects these three antibiotics have on C. difficile colonization. The purpose of this study was to elucidate the gut bacterial community changes concurrent with clearance of C. difficile colonization. We hypothesized that each colonized community had perturbation-specific susceptibilities and required specific changes to clear the pathogen. To induce a less severe perturbation, we reduced the doses of cefoperazone and streptomycin. This resulted in communities that were initially colonized to a high level (>10^6^ CFU/g feces) and then spontaneously cleared C. difficile. We found that each antibiotic resulted in unique changes in the microbiota that were associated with the persistence or clearance of C. difficile. These data further support the hypothesis that C. difficile can exploit numerous niches in perturbed communities.

## RESULTS

### Reduced doses of cefoperazone and streptomycin allowed communities to spontaneously clear C. difficile colonization.

**FIG 1:**
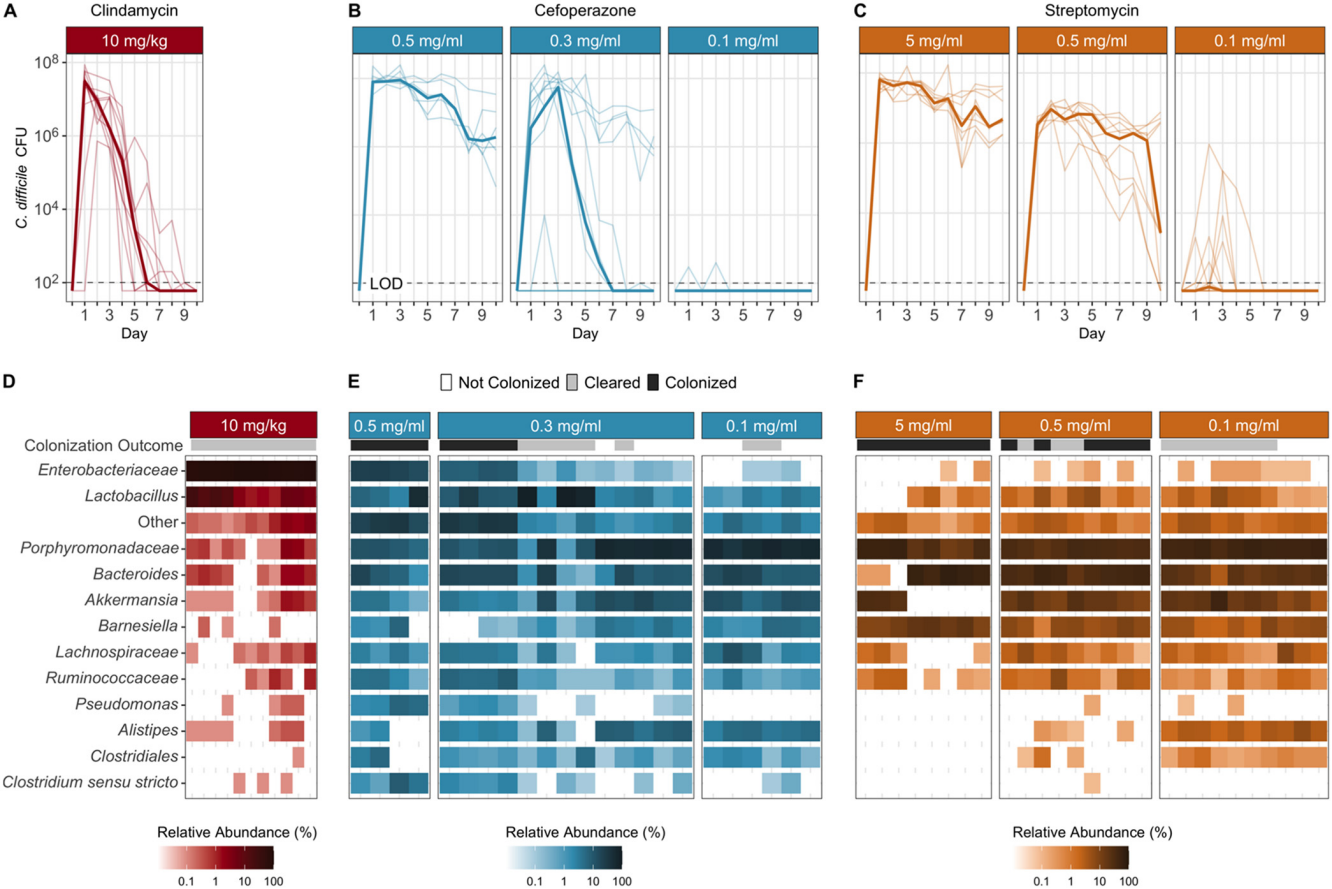
Reduced antibiotic doses permitted murine communities to be colonized by C. difficile and to spontaneously clear the C. difficile colonization. (A to C) Daily CFU counts of C. difficile in fecal samples of mice treated with clindamycin, cefoperazone, or streptomycin from time of challenge (day 0) with 10^3^ C. difficile strain 630Δerm spores through 10 days postinfection (dpi). The bold lines are the median CFU counts of the groups, and the light lines are the data for individual mice. (D to F) Relative abundances of the 12 most abundant taxonomic groups at the time of C. difficile challenge, labeled with the lowest level of classification, and all other taxonomic groups, which are combined into Other. Each column represents the data for an individual mouse. Clindamycin: 10 mg/kg, *n* = 11. Cefoperazone: 0.5 mg/ml, *n* = 6; 0.3 mg/ml, *n* = 13; 0.1 mg/ml, *n* = 6. Streptomycin: 5.0 mg/ml, *n* = 8; 0.5 mg/ml, *n* = 9; 0.1 mg/ml, *n* = 11. LOD, limit of detection.

To understand the dynamics of colonization and clearance of C. difficile, we first identified conditions which would allow colonization and clearance. Beginning with clindamycin, mice were treated with an intraperitoneal injection of the antibiotic (10 mg/kg of body weight) 1 day prior to challenge with C. difficile. All mice (*n* = 11) were colonized to a high level (median CFU = 3.07 × 10^7^) the next day and cleared the colonization within 10 days; 6 mice cleared C. difficile within 6 days (Fig. 1A). Previous C. difficile infection models using cefoperazone and streptomycin have not demonstrated clearance. Therefore, we next explored whether cefoperazone and streptomycin could permit colonization and subsequent clearance with lower doses. We began with replicating the previously established C. difficile infection models using these antibiotics (20). We treated mice with cefoperazone or streptomycin in their drinking water for 5 days (0.5 mg/ml and 5 mg/ml, respectively) and then challenged them with C. difficile. For both antibiotics, C. difficile colonization was maintained for the duration of the experiment as previously demonstrated (Fig. 1B and C) (20). Then we repeated the C. difficile challenge with reduced doses of the antibiotics (cefoperazone, 0.3 and 0.1 mg/ml; streptomycin, 0.5 and 0.1 mg/ml). For both antibiotic treatments, the lowest dose resulted in either no colonization (*n* = 8) or a transient low-level colonization (*n* = 8, median length = 1 day, median CFU/g = 2.8 × 10^3^) (Fig. 1B and C). The intermediate dose of both antibiotics resulted in a high-level colonization (median CFU/g = 3.5 × 10^6^) and half (*n* = 8 of 16) of the mice clearing the colonization within 10 days. Based on our previous research, which showed that each of these antibiotics uniquely changed the microbiota, we hypothesized that the microbiota varied across these antibiotic treatments that resulted in colonization clearance. To focus on the changes related to clearance and not antibiotic dosage, the remaining analysis aggregated mice that had C. difficile present in their stool postchallenge by whether C. difficile was detected (i.e., colonized) or not (i.e., cleared) at the end of the experiment.

### Clearance of C. difficile was associated with antibiotic-specific changes to the microbiota.

**FIG 2:**
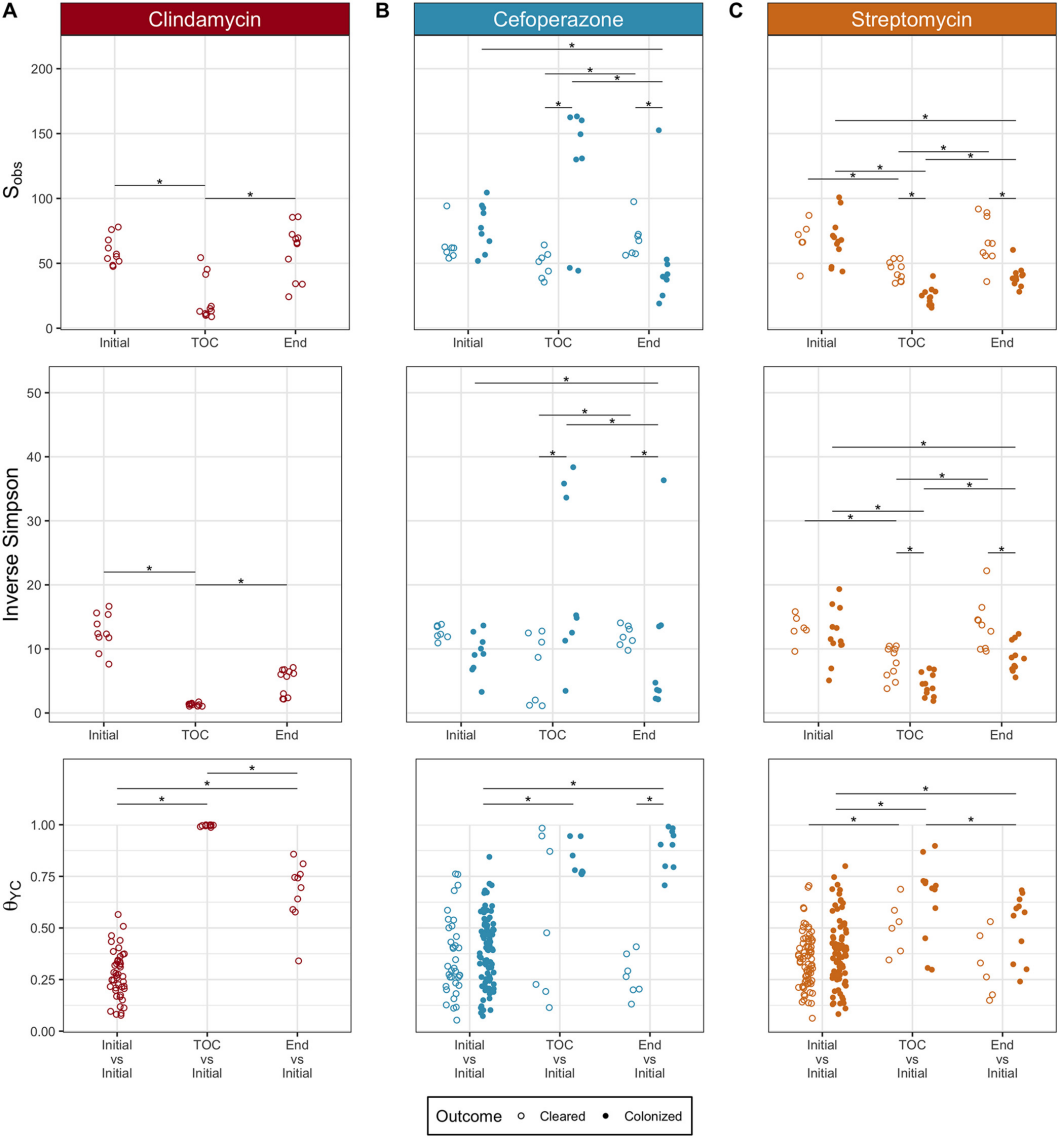
Microbiota community diversity showed antibiotic-specific trends associated with C. difficile colonization clearance. For communities colonized with C. difficile from mice treated with clindamycin (A), cefoperazone (B), and streptomycin (C), microbiota α-diversity (*S*_obs_ and inverse Simpson) and β-diversity (θ_YC_) were compared at the initial pre-antibiotic treatment state, time of C. difficile challenge (TOC), and end of the experiment. β-Diversity (θ_YC_) was compared between the initial pre-antibiotic treatment community and all other initial pre-antibiotic treatment communities treated with the same antibiotic, the initial community and the same community at the time of C. difficile challenge, and the initial community and the same community at end of the experiment. (clindamycin, *n* = 11 cleared; cefoperazone, *n* = 7 cleared, *n* = 9 colonized; streptomycin, *n* = 9 cleared, *n* = 11 colonized). *, *P < *0.05, calculated by Wilcoxon rank sum test with Benjamini-Hochberg correction.

**FIG 3:**
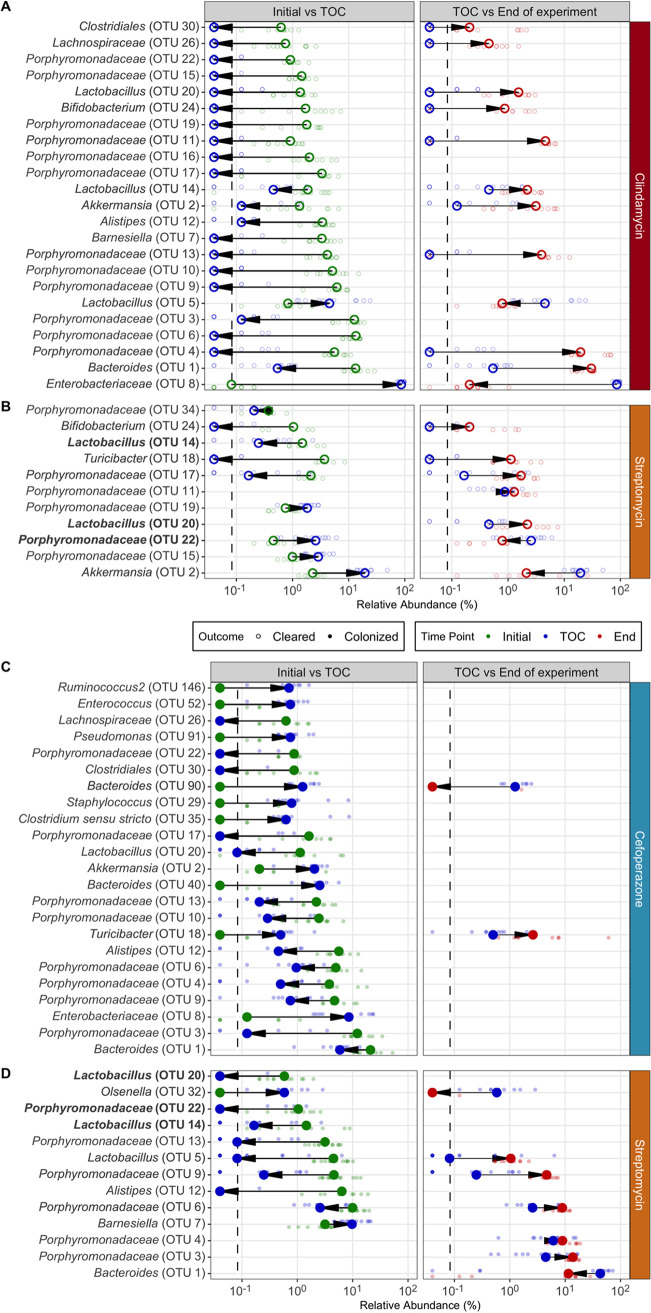
Each antibiotic had specific sets of temporal changes in OTU abundances associated with C. difficile colonization and clearance. For clindamycin (A), cefoperazone (C), and streptomycin (B, D), the differences in the relative abundances of OTUs that were significantly different between time points within each C. difficile colonization outcome (cleared, unfilled points [A, B] or colonized, filled points [C, D]) for each antibiotic treatment were identified. Dark larger points in foreground represent median relative abundances, and light smaller points in background represent relative abundances for individual mice. Lines connect points within each comparison to show differences in median values. Arrows point in the direction of the temporal change of the relative abundance. Only OTUs at time points with statistically significant differences (*P < *0.05) were plotted (calculated by Wilcoxon rank sum test with Benjamini-Hochberg correction). Bold OTUs were shared across outcomes. LOD, limit of detection; TOC, time of challenge.

Beginning with the clindamycin-treated mice, we analyzed their fecal 16S rRNA gene sequences to identify the community features related to C. difficile colonization and clearance. First, we compared the most abundant bacterial genera of the communities at the time of C. difficile challenge. The clindamycin-treated mice became dominated by relatives of *Enterobacteriaceae*, with concurrent reductions in the other abundant genera, except for populations of *Lactobacillus* (Fig. 1D, Fig. S1). These community changes permitted C. difficile to colonize all of these mice, but all of the mice were also able to clear the colonization. We next investigated how the microbiota diversity related to C. difficile clearance. Clindamycin treatment decreased the α-diversity (*P < *0.05) and similarity to the pre-clindamycin treatment community at the time of C. difficile challenge (*P < *0.05) (Fig. 2A). But it was not necessary to restore the community similarity to its initial state to clear C. difficile. Therefore, we investigated the temporal differences in the abundances of the operational taxonomic units (OTUs) between the initial untreated community and the post-clindamycin treatment community at the time of challenge and between the time of challenge and the end of the experiment. Clindamycin treatment resulted in large decreases in 21 OTUs and a bloom of relatives of *Enterobacteriaceae* (Fig. 3A). With the elimination of C. difficile, we observed a drastic reduction of the relatives of *Enterobacteriaceae* and recovery of 10 populations related to *Porphyromonadaceae*, *Bacteroides*, *Akkermansia*, *Lactobacillus*, *Bifidobacterium*, *Lachnospiraceae*, and *Clostridiales* (Fig. 3A). Thus, clindamycin reduced most of the natural community, allowing C. difficile to colonize. The recovery of only a portion of the community was associated with eliminating the C. difficile population.

10.1128/mSphere.01238-20.1 FIG S1 Relative abundances of initial microbiota of mice prior to antibiotic treatment. Initial community shows the most abundant taxa. The plot shows the relative abundances at the beginning of the experiment prior to antibiotic treatment of the 12 most abundant taxonomic groups, labeled with the lowest level of classification. All other taxonomic groups are combined into Other. Each column represents an individual mouse fecal community. Color intensity shows log_10_-transformed mean percent relative abundance. Download FIG S1, TIF file, 0.4 MB. Copyright © 2021 Lesniak et al. 2021 Lesniak et al. https://creativecommons.org/licenses/by/4.0/ This content is distributed under the terms of the Creative Commons Attribution 4.0 International license.

We applied the same analysis to the cefoperazone-treated mice to understand what community features were relevant to clearing C. difficile. Increasing the dose of cefoperazone shifted the dominant community members from relatives of the *Porphyromonadaceae*, *Bacteroides*, and *Akkermansia* to relatives of the *Lactobacillus* and *Enterobacteriaceae* at the time of challenge (Fig. 1E, Fig. S1). We saw an increase in relatives of *Enterobacteriaceae* similar to that with clindamycin. However, the cefoperazone-treated mice that had larger increases in *Enterobacteriaceae* were unable to clear C. difficile. We next investigated the differences between the cefoperazone-treated mice that cleared C. difficile and those that did not. For the communities that cleared C. difficile, diversity was maintained throughout the experiment (Fig. 2B). A subset of mice treated with cefoperazone that remained colonized experienced an increase in α-diversity, possibly driven by the decrease in highly abundant populations and increase in low-abundance populations (Fig. 1E, Fig. S2). These persistently colonized communities also had a large shift away from the initial community structure caused by the antibiotic treatment (*P < *0.05), which remained through the end of the experiment (*P < *0.05) (Fig. 2B). The α-diversity of mice treated with cefoperazone did not vary significantly by dosage (Fig. S3). These data suggested that it was necessary for cefoperazone-treated mice to become more similar to the initial, preantibiotic community structure to clear C. difficile.

10.1128/mSphere.01238-20.2 FIG S2 Cefoperazone-treated mice with increased *S*_obs_ have increased abundance of initially low abundant OTUs. Relative abundance of each OTU plotted for mice treated with cefoperazone. OTUs arranged numerically along the *x* axis. Each point is the relative abundance of a single OTU of an individual mouse. Split at *S*_obs_ = 120 separates the communities that increased in α-diversity above the α-diversity in the untreated initial communities (Fig. 2). *S*_obs_ < 120: initial, *n* = 16; time of challenge, *n* = 9; end, *n* = 15. *S*_obs_ > 120: initial, *n* = 0; time of challenge, *n* = 6; end, *n* = 1. Download FIG S2, TIF file, 1.0 MB. Copyright © 2021 Lesniak et al. 2021 Lesniak et al. https://creativecommons.org/licenses/by/4.0/ This content is distributed under the terms of the Creative Commons Attribution 4.0 International license.

10.1128/mSphere.01238-20.3 FIG S3 α-Diversity of communities from cefoperazone-treated mice that remained colonized with C. difficile was not different by antibiotic dosage. *S*_obs_ and inverse Simpson were plotted by the time point, C. difficile colonization outcome, and cefoperazone dosage and tested by Wilcoxon rank sum test with Benjamini-Hochberg correction for differences. The group with the largest difference at the time of challenge for mice that remained colonized was not significant (*P = *0.1142857). Mice that remained colonized are represented by filled points, and those that cleared colonization by unfilled points. Download FIG S3, TIF file, 0.4 MB. Copyright © 2021 Lesniak et al. 2021 Lesniak et al. https://creativecommons.org/licenses/by/4.0/ This content is distributed under the terms of the Creative Commons Attribution 4.0 International license.

**FIG 4:**
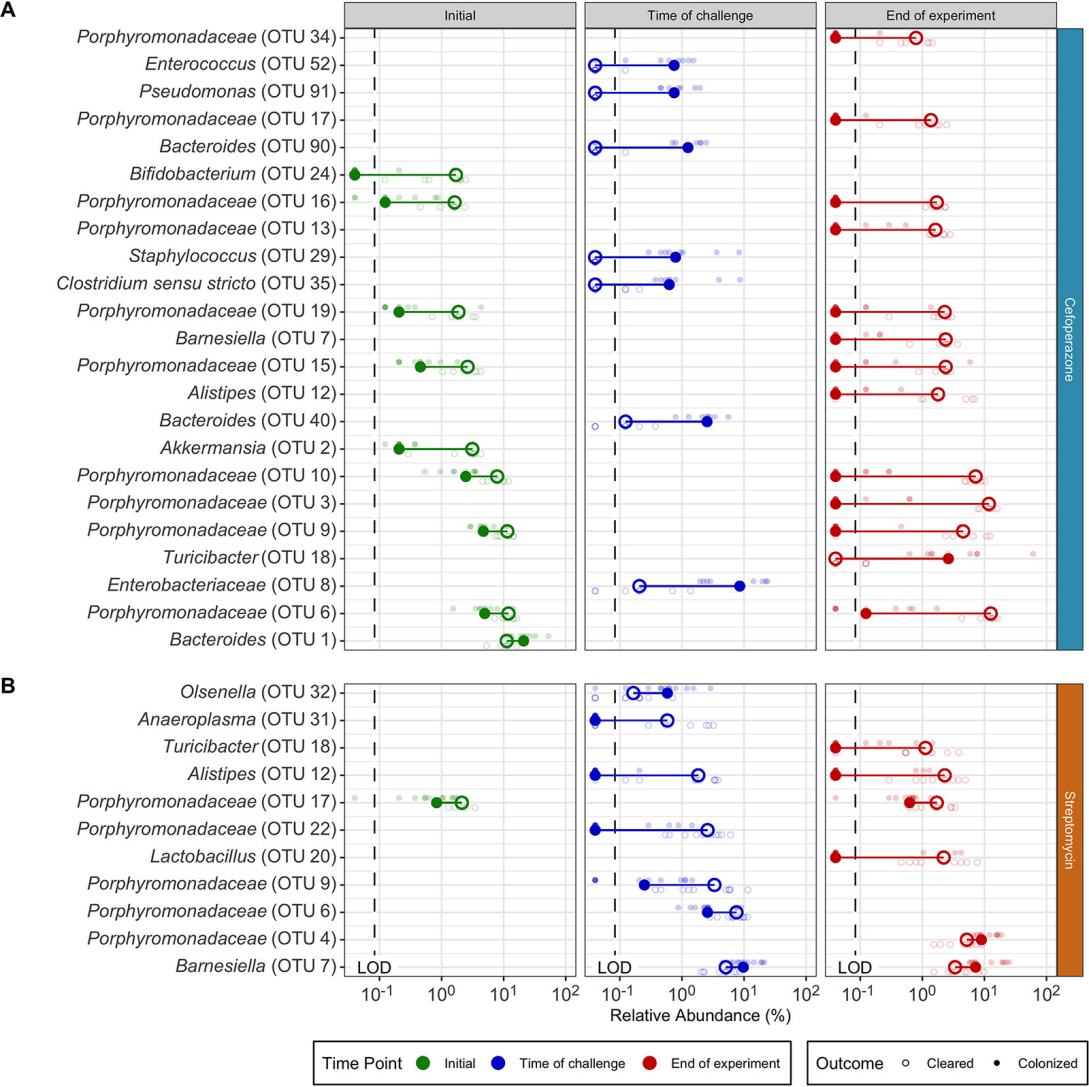
OTU abundance differences between communities that cleared C. difficile colonization and those that remained colonized are unique to each treatment. For cefoperazone (A) and streptomycin (B), the differences in the relative abundances of OTUs that were significantly different between communities that eliminated C. difficile colonization and those that remained colonized within each antibiotic treatment for each time point were identified. Dark larger points in foreground represent median relative abundances, and light smaller points in background represent relative abundances for individual mice. Lines connect points within each comparison to show differences in median values. Only OTUs at time points with statistically significant differences (*P < *0.05) were plotted (calculated by Wilcoxon rank sum test with Benjamini-Hochberg correction). LOD, limit of detection.

We next investigated the changes in OTU abundances between the communities that cleared C. difficile and those that did not to elucidate the community members involved in clearance. Communities that remained colonized were significantly enriched in facultative anaerobic populations, including *Enterococcus*, Pseudomonas, Staphylococcus, and *Enterobacteriaceae*, at the time of challenge. Communities that cleared C. difficile had significant enrichment in 10 different OTUs related to the *Porphyromonadaceae* at the end of the experiment (Fig. 4A). We were also interested in the temporal changes within each community, so we investigated which OTUs changed due to antibiotic treatment or during the C. difficile colonization. The majority of significant temporal differences in OTUs for cefoperazone-treated mice occurred in persistently colonized communities. Persistently colonized communities had a persistent loss of numerous relatives of the *Porphyromonadaceae* and increases in the relative abundances of facultative anaerobes (Fig. 3C, Fig. S4). Overall, persistent C. difficile colonization in cefoperazone-treated mice was associated with a shift in the microbiota to a new community structure that was unable to recover from the antibiotic perturbation, whereas clearance occurred when the community was capable of returning to its original structure.

10.1128/mSphere.01238-20.4 FIG S4 Temporally differing OTUs for cefoperazone-treated mice that cleared C. difficile colonization. Bold points show median relative abundances, and light points show relative abundances for individual mice. Lines connect points within each comparison to show differences in median values. Arrows point in the direction of the temporal change of the relative abundance. Only OTUs at time points with statistically significant differences (*P < *0.05) were plotted (calculated by Wilcoxon rank sum test with Benjamini-Hochberg correction). LOD, limit of detection. Download FIG S4, TIF file, 8.2 MB. Copyright © 2021 Lesniak et al. 2021 Lesniak et al. https://creativecommons.org/licenses/by/4.0/ This content is distributed under the terms of the Creative Commons Attribution 4.0 International license.

Finally, we identified the differences in C. difficile colonization for streptomycin-treated mice. Increasing the dose of streptomycin maintained the abundance of relatives of the *Porphyromonadaceae* and *Bacteroides* but reduced most of the other genera, including populations of the *Lactobacillus*, *Lachnospiraceae*, *Ruminococcaceae*, *Alistipes*, and *Clostridiales* (Fig. 1F). Both communities that cleared C. difficile and those that remained colonized had similar changes in diversity. The communities in streptomycin-treated mice became mildly dissimilar (*P < *0.05) and less diverse (*P < *0.05) with streptomycin treatment but by the end of the experiment returned to resemble the preantibiotic community (*P < *0.05) (Fig. 2C). Those communities that remained colonized had slightly lower α-diversity than those that cleared C. difficile. (*P < *0.05). Persistently colonized mice had reduced relative abundances of relatives of *Alistipes*, *Anaeroplasma*, and *Porphyromonadaceae* at the time of challenge compared to their abundances in the mice that cleared C. difficile (Fig. 4B). At the end of the experiment, the mice that were still colonized had lower abundances of *Turicibacter*, *Alistipes*, and *Lactobacillus*. Since most of the differences were reduced relative abundances in the colonized mice, we were interested to explore what temporal changes occurred between the time prior to antibiotic treatment, the time of challenge, and the end of the experiment for the communities that cleared C. difficile. The temporal changes in streptomycin-treated mice were more subtle than those observed with the other antibiotic treatments. At the time of challenge, the communities that remained colonized had reductions in 4 OTUs related to the *Porphyromonadaceae*. Those that cleared C. difficile also had changes in OTUs related to the *Porphyromonadaceae*; however, 2 populations decreased and 2 increased in abundance (Fig. 3B and D). At the end of the experiment, all communities experienced recovery of the abundance of many of the populations changed by the streptomycin treatment, but the communities that remained colonized did not recover 5 of the OTUs of *Alistipes*, *Lactobacillus*, and *Porphyromonadaceae* that were reduced by streptomycin. The differences between the streptomycin-treated mice that remained colonized and those that had been cleared of C. difficile were not as distinct as those observed with the cefoperazone treatment. The differences between colonized and cleared streptomycin-treated mice were minimal, which suggested the few differences may be responsible for the clearance. Overall, these data revealed that while there were families commonly affected across the antibiotic treatments, such as the *Porphyromonadaceae*, C. difficile clearance was associated with community and OTU differences specific to each antibiotic.

### Distinct features of the bacterial community at the time of infection predicted endpoint colonization.

**FIG 5:**
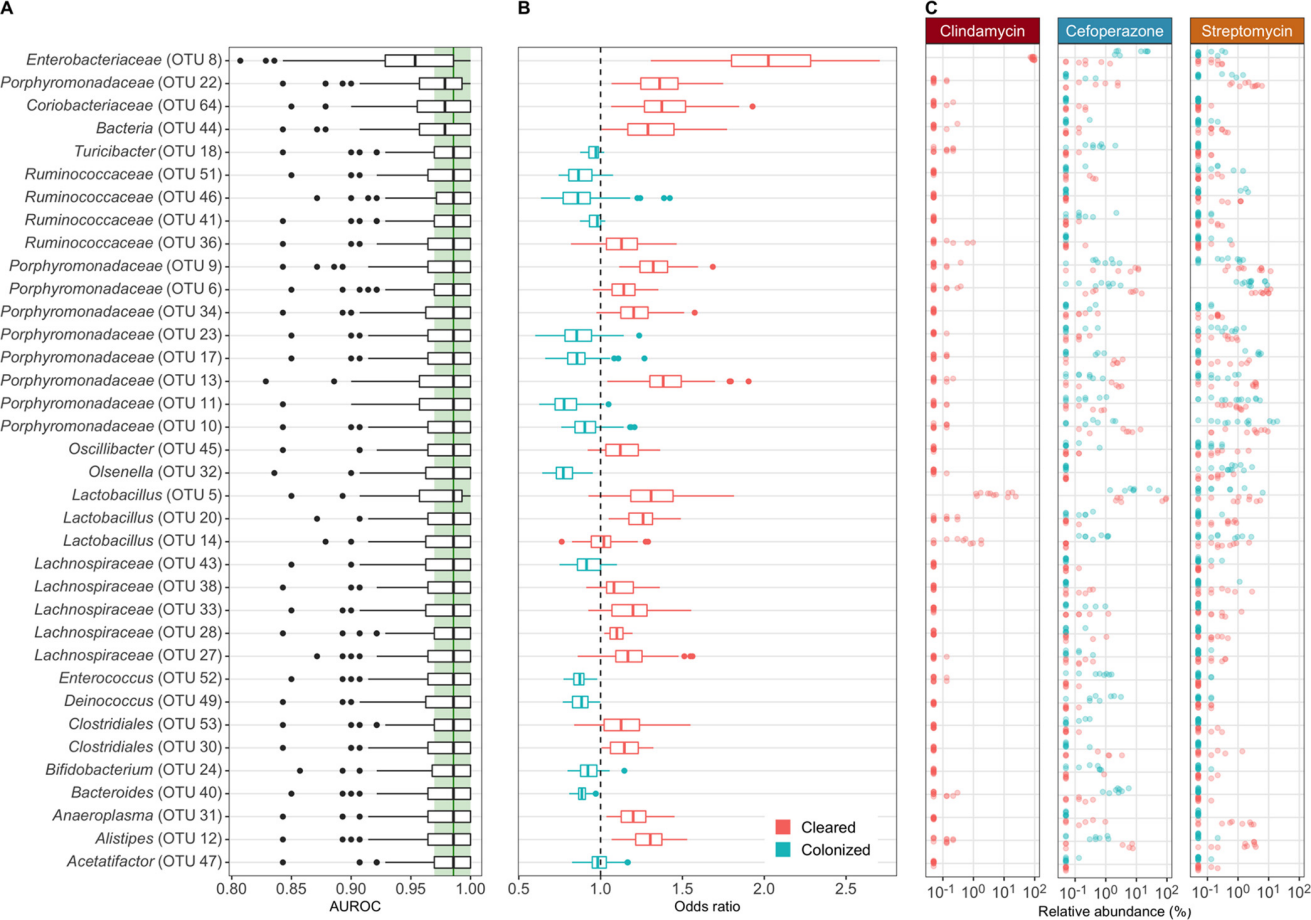
Distinct features of the bacterial community at the time of infection can classify endpoint colonization. (A) L2 logistic regression model features’ importance determined by the decrease in model performance when randomizing an individual feature. All OTUs affecting performance are shown. Light green band in the background shows the interquartile range, and dark green line shows the median AUROC of the final model with all features included. (B) Distribution of odds ratios used in L2 logistic regression model. Values above 1 indicate that the abundance predicted the community would clear C. difficile colonization (red), and values below 1 indicate that the abundance predicted C. difficile would remain colonized (blue). Feature labels and boxplots are colored to match the median odds ratios. (C) Relative abundance differences in features used by L2 logistic regression model are displayed by antibiotic treatment.

To determine whether the community composition at the time of C. difficile challenge could predict C. difficile clearance, we built a machine learning model using L2 logistic regression. We modeled all treatments together to prevent overfitting of the data and allow the model to reveal which OTUs were able to correctly predict clearance in the context of the relative abundances of other OTUs. We evaluated the predictive performance of the model using the area under the receiver operating characteristic curve (AUROC), where a value of 0.5 indicated the model was random and 1.0 indicated the model always correctly predicted the outcome. Our model resulted in a AUROC of 0.986 (interquartile range [IQR], 0.970 to 1.000), which suggested that the model was able to use the relative abundance of OTUs at the time of challenge to accurately predict colonization clearance (Fig. S5). To assess the important features, we randomly permuted each OTU feature by removing it from the training set to determine its effect on the prediction (Fig. 5A). The most important feature was an OTU related to the *Enterobacteriaceae*, whose abundance predicted clearance. This result appears to have been strongly driven by the clindamycin data (Fig. 5B and C). The remaining OTUs did not have large effects on the model performance, which suggested that the model decision was spread across many features. These results revealed that the model used the relative abundance data of the community members and the relationship between those abundances to correctly classify clearance. There were many OTUs with treatment- and outcome-specific abundance patterns that did not agree with the odds ratio of the OTU used by the model. For example, *Enterobacteriaceae* abundance influenced the model to predict clearance (Fig. 5B); however, in experiments that used cefoperazone, the communities that remained colonized had higher abundances of *Enterobacteriaceae* than the communities that cleared colonization (Fig. 5C). The model arrived at the correct prediction through the collective influence of other OTUs. Therefore, the model used different combinations of multiple OTUs and their relative abundances across treatments to predict C. difficile clearance. These data can offer a basis for hypotheses regarding the distinct combinations of bacteria that promote C. difficile clearance.

10.1128/mSphere.01238-20.5 FIG S5 Bacterial community at the time of infection can classify endpoint colonization. Classification performance of L2 logistic regression. Area under the receiver-operator curve for classifying whether the community will remain colonized based on the OTUs present at the time of C. difficile infection (day 0). Cross-validation of model was performed on half of the data to tune the model (CV AUC), and then the tuned model was tested on the held-out data (Test AUC). Download FIG S5, TIF file, 0.06 MB. Copyright © 2021 Lesniak et al. 2021 Lesniak et al. https://creativecommons.org/licenses/by/4.0/ This content is distributed under the terms of the Creative Commons Attribution 4.0 International license.

### Conditional independence networks revealed treatment-specific relationships between the community members and C. difficile during colonization clearance.

**FIG 6:**
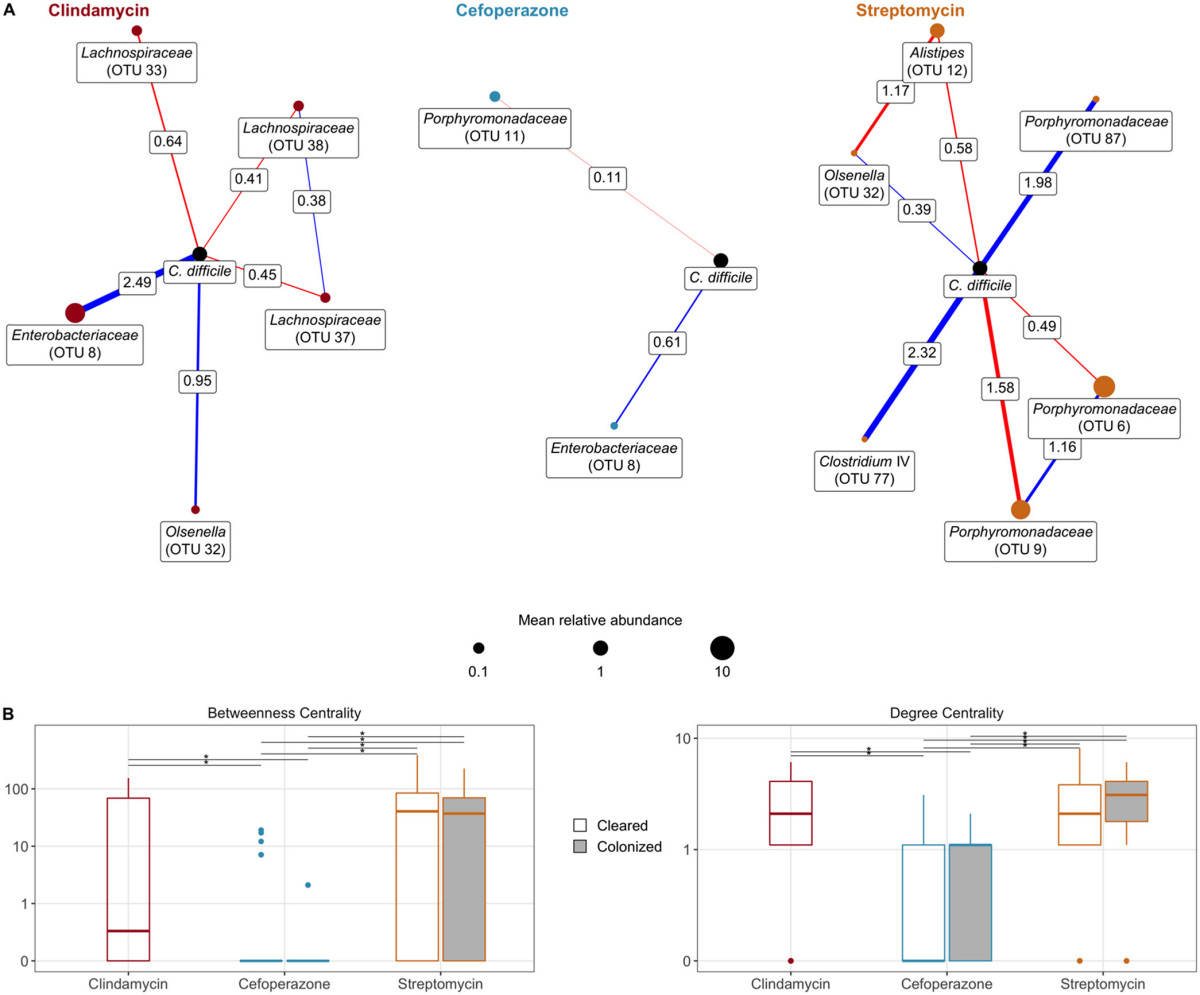
Conditional independence networks reveal treatment-specific relationships between the community and C. difficile during colonization clearance. (A) Sparse inverse covariance estimation for ecological association inference (SPIEC-EASI) networks showing conditionally independent first-order relationships between C. difficile and the community as C. difficile was cleared from the gut environment. Nodes are sized by the median relative abundance of the OTU. A red edge indicates a negative interaction, and blue indicates a positive interaction, while edge thickness indicates the interaction’s strength. (B) Network centrality measured with betweenness, i.e., how many paths between two OTUs pass through an individual, and degree, i.e., how many connections an OTU has. *, *P < *0.05, calculated by Wilcoxon rank sum test with Benjamini-Hochberg correction.

Finally, we explored the relationship between temporal changes in the community and C. difficile by building a conditional independence network for each treatment using sparse inverse covariance estimation for ecological association inference (SPIEC-EASI) (21). First, we focused on the first-order associations of C. difficile (Fig. 6A). In clindamycin-treated mice, C. difficile had positive associations with relatives of *Enterobacteriaceae*, Pseudomonas, and *Olsenella* and negative associations with relatives of the *Lachnospiraceae* and *Clostridium* XIVa. C. difficile had limited associations in cefoperazone-treated mice; the primary association was a positive one with relatives of *Enterobacteriaceae*. In streptomycin-treated mice, C. difficile had negative associations with relatives of the *Porphyromonadaceae* and positive associations with populations of the *Ruminococcaceae*, *Bacteroidetes*, *Clostridium* IV, and *Olsenella*. Next, we quantified the degree centrality, which is the number of associations between each OTU for the whole network of each antibiotic and outcome, and betweenness centrality, which is the number of associations connecting two OTUs that pass through an OTU (Fig. 6B). This analysis revealed that cefoperazone treatment resulted in networks primarily composed of singular associations with much lower degree centrality (*P < *0.05) and betweenness centrality (*P < *0.05) than the other antibiotic treatments. Communities that were treated with cefoperazone that resulted in cleared or persistent colonization had 10- to 100-fold-lower betweenness centrality values than communities treated with clindamycin or streptomycin. Collectively, these networks suggest that C. difficile colonization was affected by unique sets of OTUs in mice treated with clindamycin and streptomycin but cefoperazone treatment eliminated bacteria critical to maintaining community interactions and had few populations that associated with C. difficile.

## DISCUSSION

We have shown that different antibiotic treatments resulted in specific changes to the microbiota that were associated with C. difficile clearance. Clindamycin-treated mice became susceptible with a dominant bloom in populations related to *Enterobacteriaceae*. Clearance was associated with the resolution of the bloom and recovery of bacteria that were reduced by the antibiotic treatment. Cefoperazone-treated mice became susceptible with the expansion of numerous facultative anaerobes. Communities with a sustained presence of these facultative anaerobes were unable to recover from the initial antibiotic perturbation or clear the colonization, whereas communities that returned to their initial community structure were able to clear C. difficile colonization. Streptomycin-treated mice became susceptible with fewer and smaller changes than mice that received the other treatments. The communities that cleared colonization had slightly higher α-diversity than those that remained colonized. Additionally, all communities in mice treated with streptomycin had similar numbers of OTUs changing through the experiment, but the specific OTUs were different for each outcome. These observations support our hypothesis that each colonized community has antibiotic-specific changes that create unique conditions for C. difficile colonization and that specific changes within each community are required to clear C. difficile.

Previous studies have identified microbiota associated with reduced C. difficile colonization in either a set of closely related murine communities or collectively across many different susceptible communities (11, 15, 22). Bacteria from these studies have since been tested in C. difficile infection models. These experiments either showed decreased colonization but not elimination of C. difficile (11, 23) or only demonstrated elimination in the model that was developed (15). Rather than looking for similarities across all susceptible communities, we explored the changes that were associated with C. difficile clearance for each antibiotic. Even though these mice all came from the same breeding colony and had similar initial microbiomes, C. difficile clearance was associated with antibiotic-specific changes in community diversity, OTU abundances, and associations between OTUs. Our data suggest that the set of bacteria necessary to restore colonization resistance following one antibiotic perturbation may not be effective for all antibiotic perturbations. We have developed this modeling framework starting from a single mouse community. It should also be relevant when considering interpersonal variation among humans (24).

Recent studies have begun to uncover how communities affect C. difficile colonization (17–20, 24). We attempted to understand the general trends in each antibiotic treatment that lead to clearance of C. difficile. We categorized the general changes and microbial relationships of these experiments into three models. First, a model of temporary opportunity characterized by the transient dominance of a facultative anaerobe that permits C. difficile colonization but in which C. difficile is not able to persist, as with clindamycin treatment. We hypothesize that this susceptibility is due to a transient repression of community members and that interventions that further perturb the community may worsen the infection. Time alone may be sufficient for the community to clear colonization (15, 22, 25), but treating the community with an antibiotic or the bowel preparation for an FMT (26, 27) may prolong susceptibility by eliminating protective functions or opening new niches. Second, a model of an extensive opportunity characterized by a significant perturbation that leads to a persistent increase in facultative anaerobes and exposes multiple niches, as with cefoperazone treatment. These communities appear to have been severely depleted of multiple critical community members and are likely lacking numerous protective functions (20). We hypothesize that multiple niches are made available for C. difficile to colonize through reduced populations of bacteria that produce inhibitory molecules or compete for either nutrients or space, increasing available resources. In this scenario, community restoration will require transplantation with microbes that provide adequate diversity and abundance to outcompete and occupy all the exposed niches. If this diversity is not provided through a single FMT, multiple FMTs (28, 29) or transplant of an enriched fecal community (30) may be necessary to recover the microbiota enough to outcompete C. difficile for the nutrient niches and replace the missing protective functions. Third, a model of a specific opportunity characterized by a perturbation that only affects a select portion of the microbiota, leading to small changes in relative abundances and a slight decrease in diversity, opening a limited niche for C. difficile to colonize, as with streptomycin treatment. We hypothesize that a few specific bacterial species with key inhibitory functions would be necessary to recolonize the exposed niche space and eliminate C. difficile colonization (13, 17). A fecal microbiota transplant may contain the bacterial diversity needed to fill the open niche space and help supplant C. difficile from the exposed niche of the colonized community. Analyzing each of these colonization models individually allowed us to understand how each may clear C. difficile colonization.

Future investigations can further identify the exposed niches of susceptible communities and the requirements to clear C. difficile colonization. One common theme for susceptibility across treatments was the increased abundance of facultative anaerobes. These blooms of facultative anaerobes could be attributed to the loss of the indigenous obligate anaerobes with antibiotic treatment (31, 32). However, it is unclear what prevents the succession from the facultative anaerobes back to the obligate anaerobes in cefoperazone-treated mice. Future studies should investigate the relationship between facultative anaerobe blooms and susceptibility to colonization, as well as interventions to recover the obligate anaerobes. Another aspect to consider in future experiments is C. difficile strain specificity. Other strains may fill different niche spaces and fill other community interactions (33–35). For example, more virulent strains, such as C. difficile VPI 10463, may have a greater effect on the gut environment, since they produce more toxin and drive a stronger immune response (15, 35, 36). Those differences could lead to greater increases in inflammatory conditions and further increase populations that thrive under these conditions, such as *Enterobacteriaceae*, and thus change the requirements to clear C. difficile (31, 37, 38). Finally, we have shown that the functions found in communities at peak colonization were antibiotic specific (20). We found that the bacterial population changes associated with C. difficile clearance were antibiotic specific. It is unknown how the community functions contributing to C. difficile clearance compare across antibiotics. It is possible that we observed different changes in the bacterial populations but the functions eliminating C. difficile were conserved. Additionally, it is unclear how specific these functions are to the OTUs we observed. It is possible that phylogenetically diverse OTUs have similar functional potential, as well as phylogenetically similar OTUs having specific functions. Examining the changes in transcription and metabolites during clearance will help define the activities necessary to clear C. difficile and whether they are specific to the perturbation. This information will build upon the community differences presented in this study and move us closer to elucidating how the microbiota clears C. difficile colonization and to developing targeted therapeutics.

We have shown that mice became susceptible to C. difficile colonization after three different antibiotic treatments and then differed in their ability to clear the colonization. These experiments have shown that each antibiotic treatment resulted in different community changes that led to C. difficile clearance. These differences suggest that a single mechanism of infection and one treatment for all C. difficile infections may not be appropriate. While our current use of FMT to eliminate CDI is highly effective, it does not work in all patients and has even resulted in adverse consequences (7–10). The findings in this study may help explain why FMTs may be ineffective. Although an FMT transplants a whole community, it may not be sufficient to replace the missing community members or functions to clear C. difficile. Alternatively, the FMT procedure itself may disrupt the natural recovery of the community. The knowledge of how a community clears C. difficile colonization will advance our ability to develop targeted therapies to manage CDI.

## MATERIALS AND METHODS

### Animal care.

Five- to 8-week-old male and female C57BL/6 mice were obtained from a single breeding colony. Mice were housed in cages of 2 to 5 mice maintained under specific-pathogen-free (SPF) conditions at the University of Michigan animal facility. Each experimental treatment used 6 to 11 mice and was repeated 2 to 4 times. All mouse protocols and experiments were approved by the University Committee on Use and Care of Animals at the University of Michigan and completed in agreement with approved guidelines.

### Antibiotic administration.

Mice were given one of three antibiotics, cefoperazone, clindamycin, or streptomycin. Cefoperazone (0.5, 0.3, or 0.1 mg/ml) and streptomycin (5, 0.5, or 0.1 mg/ml) were delivered via drinking water for 5 days. Clindamycin (10 mg/kg) was administered through intraperitoneal injection.

### C. difficile challenge.

Mice were returned to untreated drinking water for 24 h before being challenged with C. difficile strain 630Δerm spores. C. difficile spores were aliquoted from a single spore stock stored at 4°C. Spore concentration was determined 1 week prior to the day of challenge (39). An amount of 10^3^ C. difficile spores was administered by oral gavage into each mouse. Once the gavages were completed, the remaining spore solution was serially diluted and plated to confirm the spore concentration that was delivered.

### Sample collection.

Fecal samples were collected on the day antibiotic treatment was started, the day of C. difficile challenge, and the following 10 days. For the day of challenge and beyond, a fecal sample was also collected and weighed. Under anaerobic conditions, a fecal sample was serially diluted in anaerobic phosphate-buffered saline and plated on TCCFA plates (47). After 24 h of anaerobic incubation at 37°C, the number of CFU was determined (40).

### DNA sequencing.

Total bacterial DNA was extracted from each fecal sample using the Mo Bio PowerSoil high-throughput (HTP) 96-well soil DNA isolation kit. We created amplicons of the 16S rRNA gene V4 region and sequenced them using an Illumina MiSeq as described previously (41).

### Sequence curation.

Sequences were processed using mothur (version 1.43.0) as previously described (41, 42). Briefly, we used a 3% dissimilarity cutoff to group sequences into operational taxonomic units (OTUs). We used a naive Bayesian classifier with the Ribosomal Database Project training set (version 16) to assign taxonomic classifications to each OTU (43). With the fecal samples, we also sequenced a mock community with a known community composition and their true 16s rRNA gene sequences. We processed this mock community along with our samples for sequence curation and found an error rate of 0.019%.

### Statistical analysis and modeling.

Diversity comparisons were calculated in mothur. To compare α-diversity metrics, we calculated the number of OTUs (*S*_obs_) and the inverse Simpson diversity index. To compare across communities, we calculated dissimilarity matrices based on the metric of Yue and Clayton (44). All calculations were made by rarifying samples to 1,200 sequences per sample to limit biases due to uneven sampling. OTUs were subsampled to 1,200 counts per sample, and the remaining statistical analysis and data visualization were performed in R (version 3.5.1) with the tidyverse package (version 1.3.0). The levels of significance of pairwise comparisons of α-diversity (*S*_obs_ and inverse Simpson), β-diversity (θ_YC_), OTU abundance, and network centrality (betweenness and degree) were calculated by the pairwise Wilcoxon rank sum test, and then *P* values were corrected for multiple comparisons with a Benjamini-Hochberg adjustment for a type I error rate of 0.05 (45). Logistic regression models were constructed with OTUs from all day 0 samples, using half of the samples to train and the other half to test the model. The model was developed from the caret R package (version 6.0-85) and previously developed machine learning pipeline (46). For each antibiotic treatment, conditional independence networks were calculated from the day 1 through 10 samples of all mice initially colonized using sparse inverse covariance estimation for ecological association inference (SPIEC-EASI) methods from the SpiecEasi R package after optimizing lambda to 0.001 with a network stability of between 0.045 and 0.05 (version 1.0.7) (21). The network centrality measures degree and betweenness were calculated on whole networks using functions from the igraph R package (version 1.2.4.1).

### Data availability.

Scripts necessary to reproduce our analysis and this paper are available in an online repository (https://github.com/SchlossLab/Lesniak_Clearance_mSphere_2021).

All 16S rRNA gene sequence data and associated metadata are available through the Sequence Read Archive via accession number PRJNA674858.
