# Supplementary figures and images for: Clearance of Clostridioides difficile Colonization Is Associated with Antibiotic-Specific Bacterial Changes
Source: mSphere. 2021 May 5;6(3):e01238-20. doi: 10.1128/mSphere.01238-20 (PMC8103992; doi:10.1128/mSphere.01238-20)

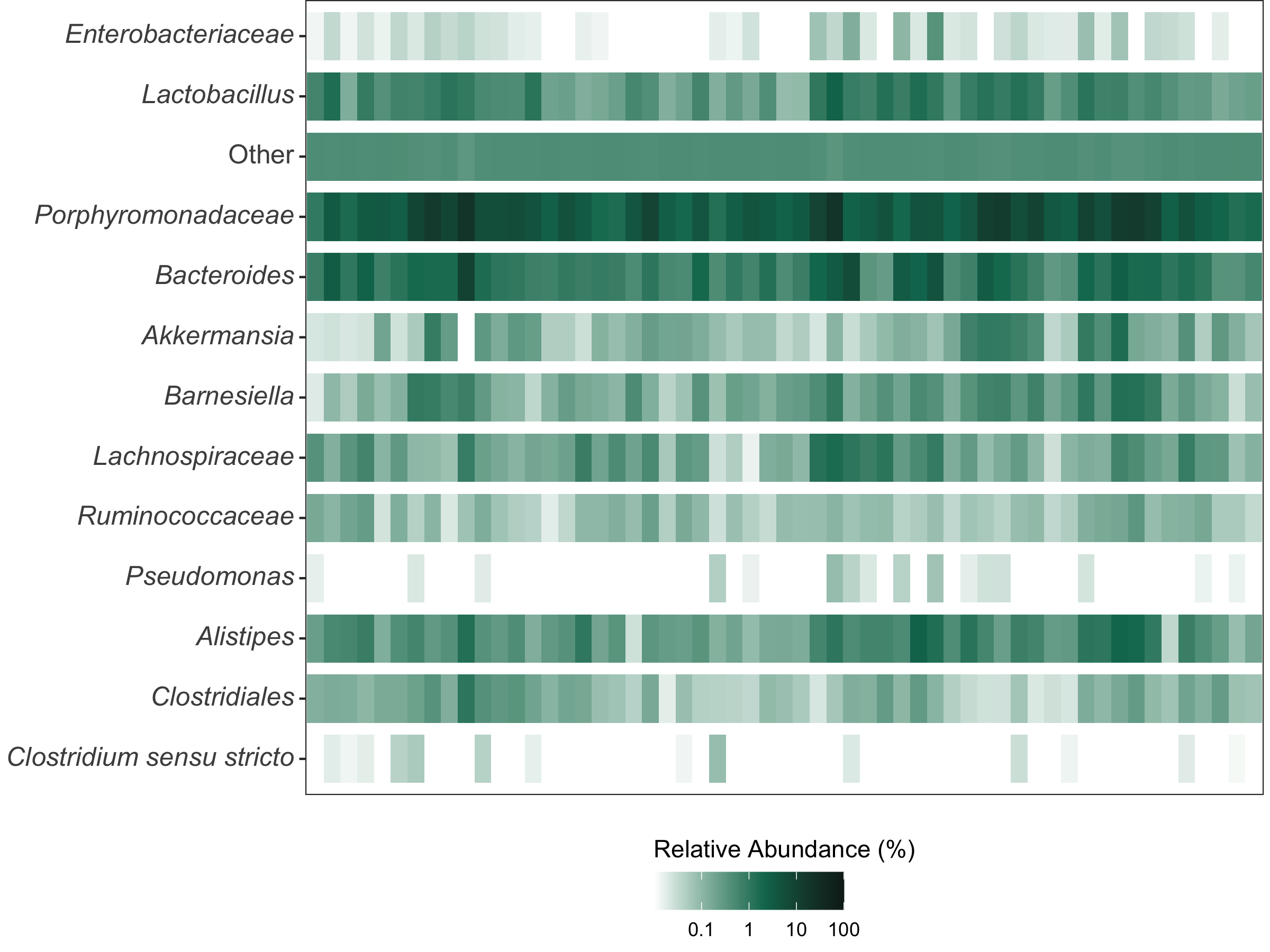

Supplement: FIG S1 [file mSphere.01238-20-sf001.tif]

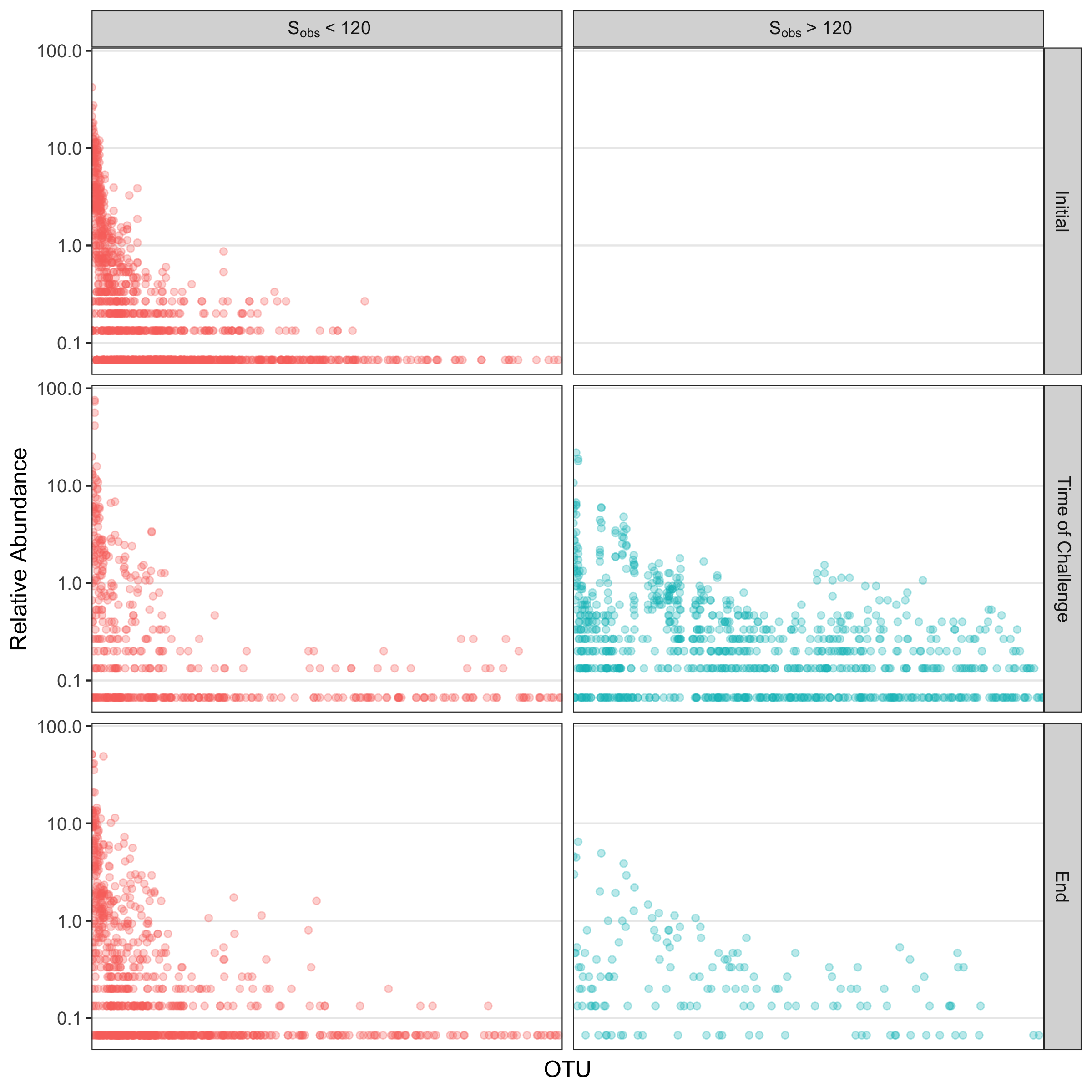

Supplement: FIG S2 [file mSphere.01238-20-sf002.tif]

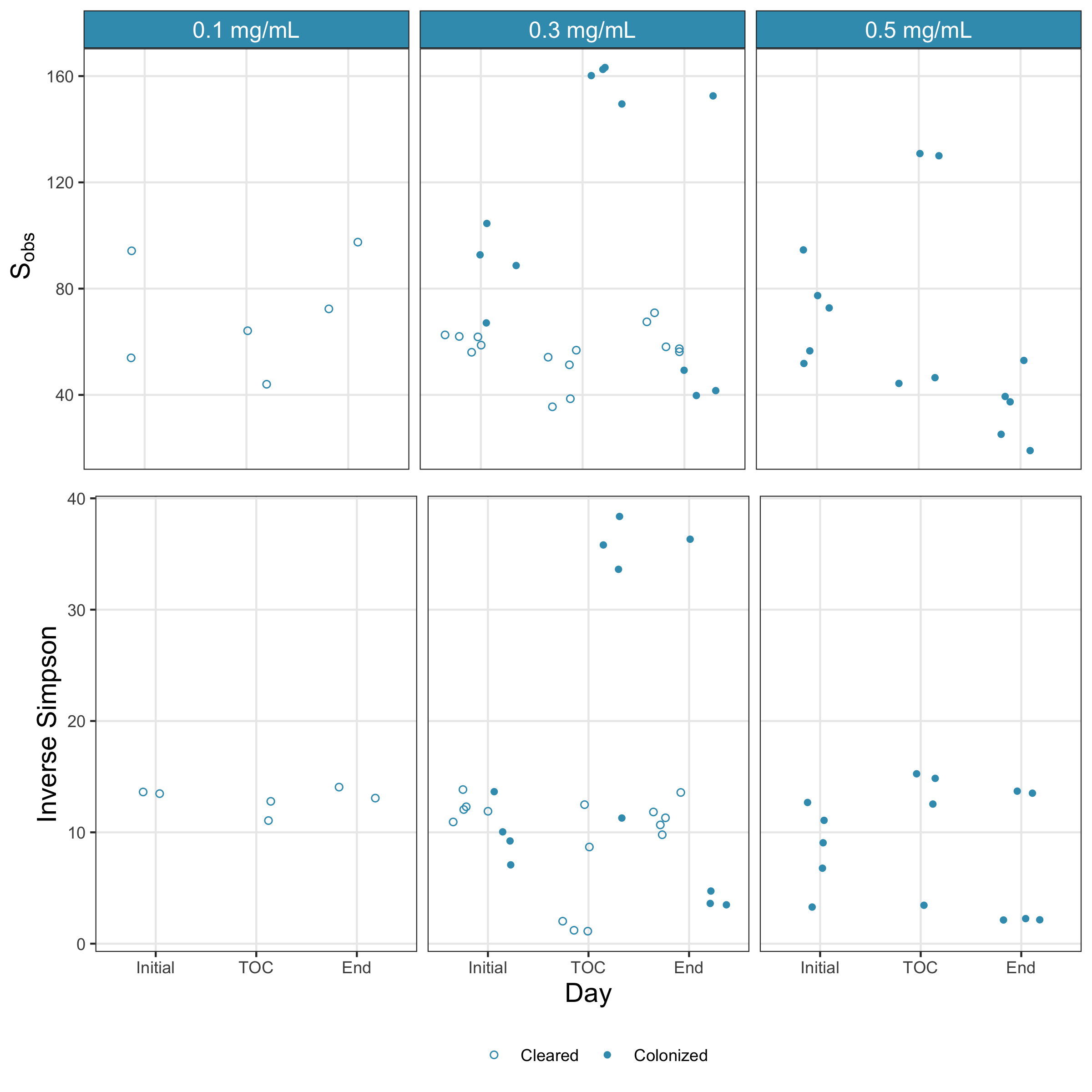

Supplement: FIG S3 [file mSphere.01238-20-sf003.tif]

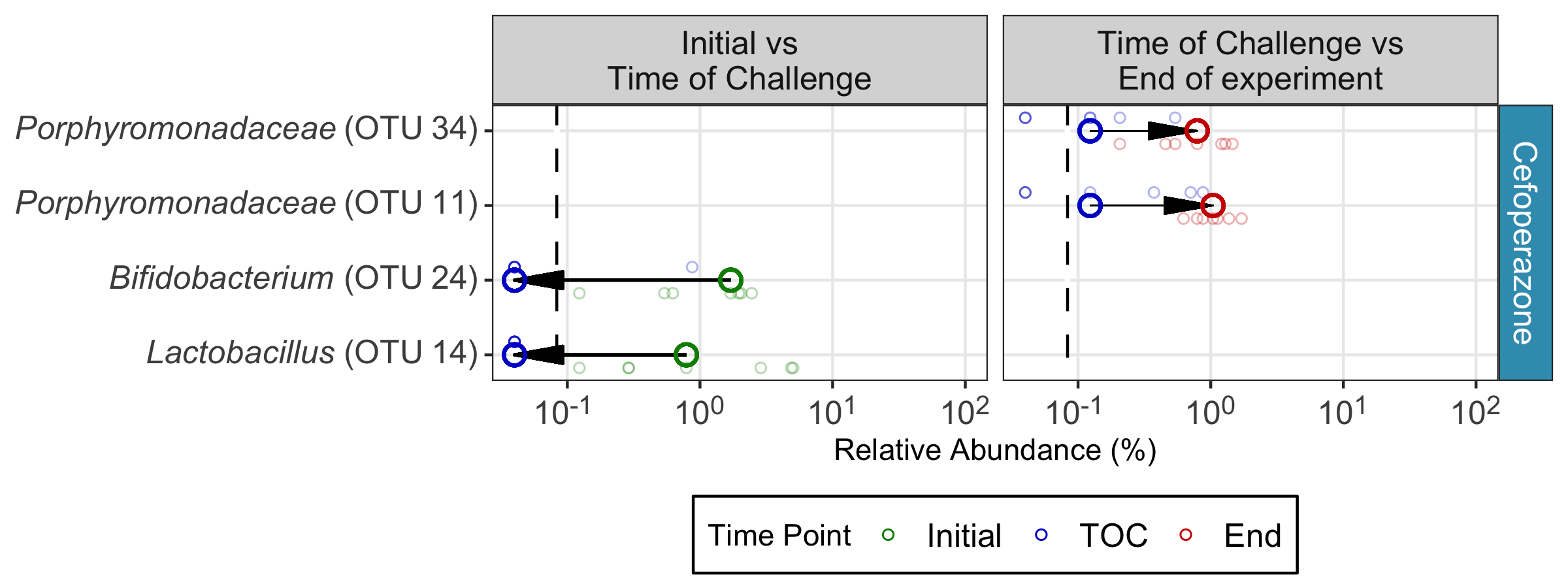

Supplement: FIG S4 [file mSphere.01238-20-sf004.tif]
